# Global trends and hotspots of macrophage-related research in intervertebral disc degeneration from 2005 to 2025: a bibliometric and visualized analysis

**DOI:** 10.3389/fimmu.2026.1856147

**Published:** 2026-07-28

**Authors:** Muhuan Liu, Jinglan He, Jizhen Zhou

**Affiliations:** 1 Hebei University of Engineering, Handan, Hebei, China; 2 Affiliated Hospital of Hebei Engineering University, Handan, Hebei, China

**Keywords:** bibliometrics, immune microenvironment, intervertebral disc degeneration, macrophage, macrophage polarization, tissue regeneration

## Abstract

**Background:**

Macrophage-mediated imbalances in the immune microenvironment are critical to the pathogenesis of intervertebral disc degeneration (IVDD). Consequently, targeting macrophage polarization to remodel the degenerative microenvironment and promote tissue repair has emerged as a major research hotspot. However, systematic bibliometric analyses of this interdisciplinary field remain scarce. This study utilizes bibliometric methods to elucidate the current landscape, intellectual base, and research frontiers of macrophage involvement in IVDD, providing quantitative data to support the development of novel immunotherapies.

**Methods:**

We retrieved literature investigating the role of macrophages in IVDD, published between 2005 and 2025, from the WoSCC and Scopus databases. Following deduplication and data standardization, 550 publications were included. We employed CiteSpace, VOSviewer, and the R package “bibliometrix” to conduct visual analyses of contributing countries, institutions, journals, and authors. Additionally, we mapped keyword co-occurrence and citation burst networks to quantitatively evaluate evolutionary trends and research hotspots.

**Results:**

Publications in this field have surged significantly since 2021. China and USA are the primary contributors, although multinational collaborative networks remain limited. *Frontiers in Immunology* and *Spine* represent the core journals driving this discipline. Keyword clustering reveals five primary research domains: pathological mechanisms, ECM regulation, tissue regeneration, multi-omics, and clinical diagnostics. Furthermore, citation burst analyses identify “single-cell sequencing,” “oxidative stress,” “injectable hydrogels,” and “tissue regeneration” as current research epicenters.

**Conclusion:**

IVDD research has undergone a critical paradigm shift, transitioning from traditional morphological observation to precision immunometabolic intervention. Currently, the research frontier focuses on integrating high resolution single cell transcriptomics with advanced nanoscale delivery systems, such as lipid nanoparticles and functionalized hydrogels, to proactively reprogram macrophage phenotypes. Ultimately, this study provides a robust quantitative framework to guide the clinical development of targeted nonsurgical immunotherapies and regenerative strategies for discogenic pain.

## 1 Introduction

IVDD is the primary pathological mechanism underlying low back pain. According to the 2021 GBD study, 619 million people globally suffered from low back pain in 2020, with projections reaching 843 million by 2050. As the leading global cause of YLDs, IVDD imposes a severe socioeconomic burden (1, 2). Traditional etiologies attribute IVDD to physiological wear from aging, abnormal mechanical stress, and genetic susceptibility. However, recent pathological paradigms have shifted, revealing that an imbalanced local immune microenvironment critically drives IVDD initiation and progression (3). Under normal physiological conditions, intervertebral discs are highly “immune-privileged.” This status is maintained by the physical barriers of the cartilage endplate and annulus fibrosus, alongside the stable expression of immunosuppressive molecules (4). When the degenerative cascade begins or cumulative mechanical microtrauma occurs, this immune barrier is compromised. Consequently, the inner NP tissue is directly exposed to the host immune system, triggering robust autoimmune responses and inducing the abnormal chemotactic infiltration of peripheral immune cells (5).

Within the complex immune cascade of IVDD, macrophages serve as a crucial hub connecting the innate and adaptive immune networks (6). Depending on microenvironmental signals, macrophages polarize into either a pro-inflammatory M1 or an anti-inflammatory/pro-reparative M2 phenotype. This polarization precisely regulates intrinsic disc cell fate and ECM metabolic homeostasis (7, 8). Specifically, the accumulation of M1 macrophages leads to the release of abundant pro-inflammatory mediators, such as IL-1β and TNF-α. These mediators directly exacerbate ECM degradation and induce irreversible NPC senescence and apoptosis (9, 10). Simultaneously, the progressive accumulation of abnormal metabolites (e.g., excess cholesterol) or damage-associated molecular patterns (DAMPs, e.g., HMGB1) further accelerates M1 polarization. This establishes a pathogenic positive feedback loop within the local degenerative microenvironment (11, 12). Extensive clinical sample analyses corroborate that macrophage infiltration and M1 polarization ratios are significantly elevated in tissues from patients with severe lumbar disc herniation and Modic changes (13). Conversely, targeted macrophage reprogramming—proactively inducing a phenotypic shift toward the tissue-reparative M2 subset—has emerged as a cutting-edge strategy to reverse this immune imbalance (14). Innovative therapies using natural small-molecule compounds (e.g., nimbolide), mesenchymal stem cell-derived exosomes, and exercise-mimetic exosomes can precisely modulate these polarization phenotypes. These approaches demonstrate substantial translational potential for decelerating disc degeneration and promoting matrix regeneration (15–17).

While numerous studies have explored the correlation between macrophages and IVDD pathology, a systematic bibliometric analysis of this specific immunological frontier is still lacking. Objective quantitative mapping is needed to accurately assess the global research landscape, intellectual base, and evolution of hotspots in this field. Existing bibliometric analyses predominantly address broader inflammatory mechanisms in IVDD (18) or general immune cell functions (19). They fail to isolate and focus on “macrophage-mediated microenvironment remodeling” as a distinct and central research trajectory.

To address this critical knowledge gap, our study employed advanced visualization software, including VOSviewer and CiteSpace, to systematically analyze the global scientific literature on the role of macrophages in IVDD from 2005 to 2025. To avoid analytical bias from missing essential metadata (e.g., citation linkages, institutional affiliations), we excluded the PubMed database, as its data structure is not optimized for mainstream bibliometric software. Instead, we used the Web of Science Core Collection and Scopus as our primary data sources, given their comprehensive and highly standardized citation data. This study aims to delineate the multinational collaboration networks, core knowledge flow pathways, and emerging research frontiers. Ultimately, we provide robust data support and forward-looking guidance for researchers exploring novel therapeutic strategies targeting the IVDD immune microenvironment.

## 2 Methods

### 2.1 Data sources and search strategy

We retrieved literature from the WoSCC and Scopus databases for the period between January 1, 2005, and December 31, 2025. For Scopus, the search query was: TITLE-ABS-KEY ((“intervertebral disc degeneration” OR “intervertebral disk degeneration” OR “degenerative disc disease” OR “disc degeneration” OR IVDD) AND (macrophage* OR “macrophage polarization” OR “M1 polarization” OR “M2 polarization”)). For WoSCC, the query was: TS=((“intervertebral disc degeneration” OR “intervertebral disk degeneration” OR “degenerative disc disease” OR “disc degeneration” OR IVDD) AND (macrophage* OR “M1 polarization” OR “M2 polarization”)).

We excluded PubMed from this study for three methodological reasons. First, exported PubMed records lack the essential metadata (such as citation frequencies and reference networks) required for bibliometric analysis. Second, its classification system frequently incorporates non-research items and occasionally omits institutional affiliations. Finally, mainstream visualization tools like VOSviewer and CiteSpace are algorithmically optimized specifically for WoSCC and Scopus data structures. While limiting our data sources exclusively to WoSCC and Scopus ensures algorithmic compatibility and prevents analytical errors arising from metadata deficiencies, we acknowledge that this methodological trade-off inherently introduces selection bias. Specifically, the exclusion of PubMed may result in the underrepresentation of purely clinical trials, localized medical case reports, and regional studies. Nevertheless, to construct robust and accurate citation linkage networks, this database restriction was deemed a necessary methodological step, with its broader implications further discussed in the Limitations section.

### 2.2 Literature screening and data cleaning

**Figure 1:**
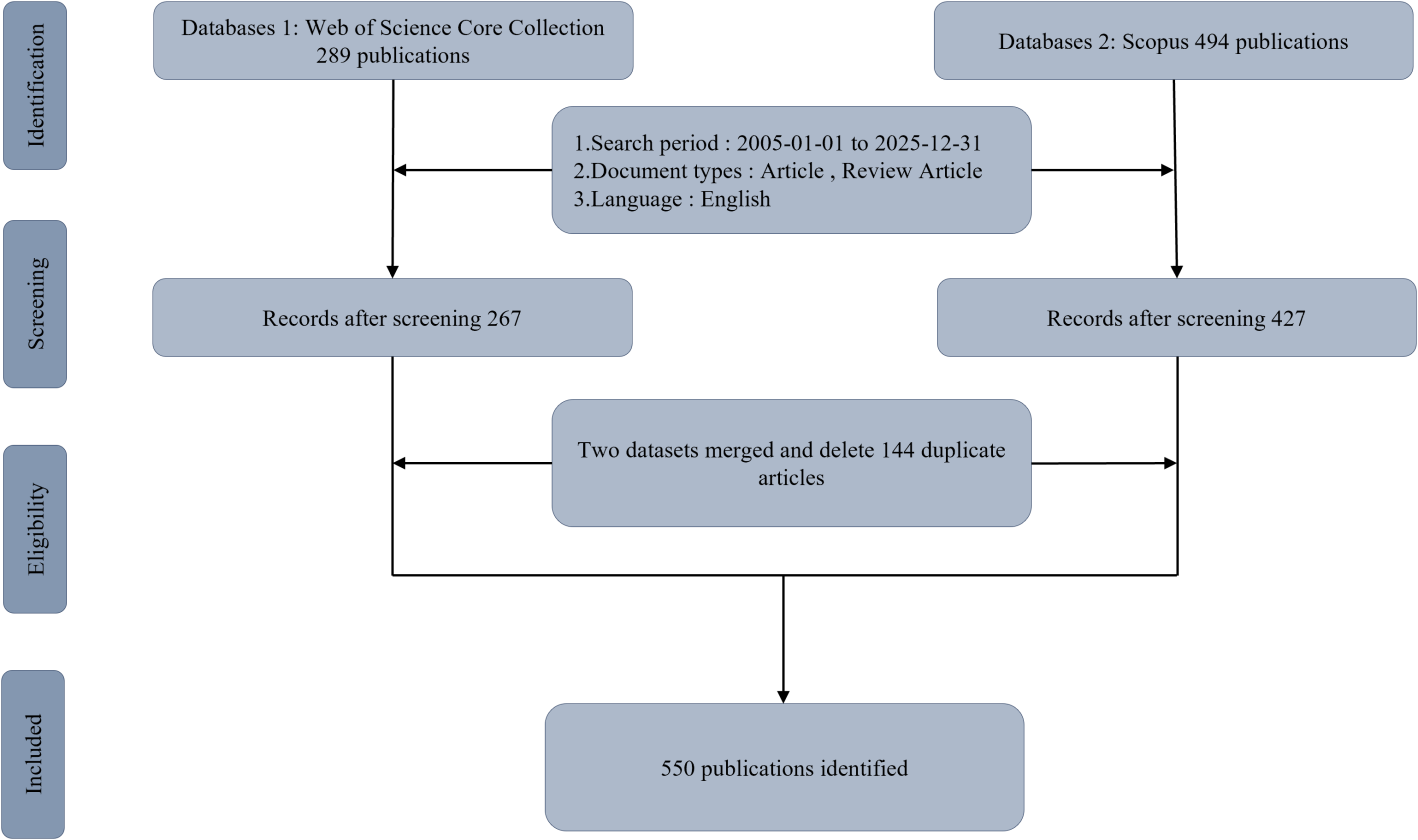
Flowchart of the retrieval and screening process.

Our initial search identified 783 records (Scopus: 494; WoSCC: 289). After filtering for the publication years 2005–2025, we retained 733 articles. We then limited the document types strictly to original articles and reviews, which resulted in 709 records. Finally, restricting the publication language to English yielded 694 records (Scopus: 427; WoSCC: 267) (**Figure 1**).

To eliminate overlapping records between the two databases, we merged the datasets and performed data cleaning and standardization using the inherent deduplication function in CiteSpace (version 6.4.R1). Ultimately, we included 550 deduplicated records in our final visualized analysis (**Figure 1**).

### 2.3 Bibliometric and visualized analysis

We performed basic statistical analyses of the data using Microsoft Excel 2021. To map global publication and citation trends, national collaboration networks, and highly cited references, we utilized the “bibliometrix” package in R Gui (version 4.5.2). We employed VOSviewer (version 1.6.20) to construct co-occurrence networks for core authors and high-frequency keyword clusters. Furthermore, we used CiteSpace (version 6.4.R1) to generate keyword evolution timelines, compute the betweenness centrality of key nodes, construct journal dual-map overlays, and execute citation and keyword burst analyses.

## 3 Results

### 3.1 Global annual publication and citation trends

**Figure 2:**
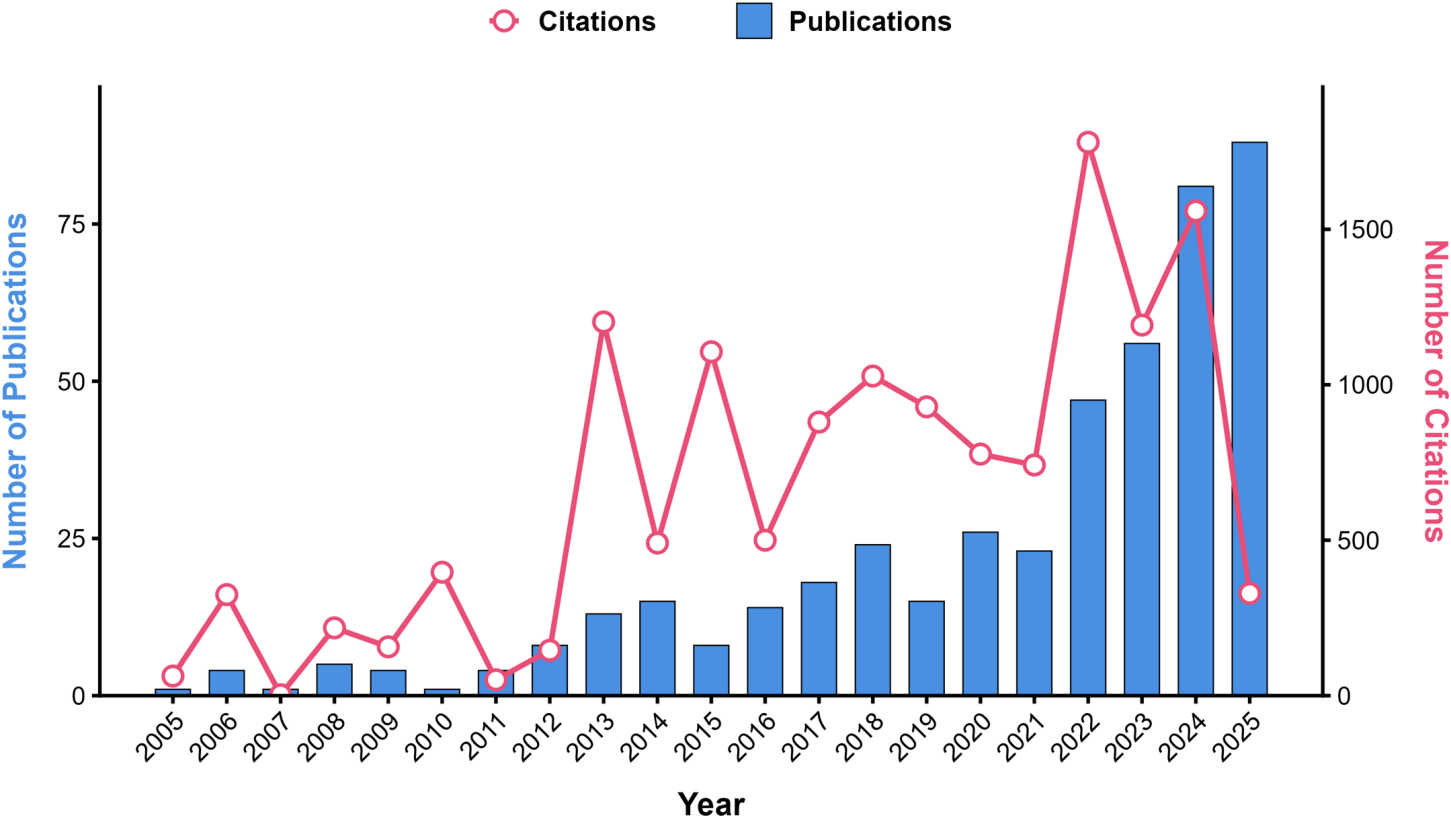
Annual publication and citation trends of macrophage-related research in IVDD from 2005 to 2025. The blue bars represent the annual number of publications (left y-axis), and the pink line with open circles indicates the annual number of citations (right y-axis).

As illustrated in **Figure 2**, research on the role of macrophages in IVDD exhibited a distinct, phased growth trend from 2005 to 2025. Based on the annual publication volume (blue bars), the evolution of this field can be categorized into three stages. The initial exploration phase (2005–2012) featured minimal output, averaging fewer than 10 articles per year. This indicates that mechanistic investigations into macrophages in IVDD were still in their infancy. The steady development phase (2013–2021) showed a fluctuating but upward trend, yielding 10 to 26 articles annually. Notably, the annual citation count (pink line) exhibited several significant peaks during this period, including over 1,100 citations in 2013 and over 1,000 in 2015. These peaks reflect the emergence of high-impact foundational literature that linked the immune microenvironment to IVDD, providing crucial theoretical support for subsequent mechanistic studies.

A rapid growth phase commenced in 2022. The annual number of publications surged between 2022 and 2025, surpassing 80 articles in 2024 and reaching an all-time high in 2025. Correspondingly, the annual citation count reached its historical maximum of approximately 1,800 in 2022, highlighting a highly concentrated period of academic attention. Although publication volumes remained high through 2024 and 2025, the citation count for 2025 naturally declined. This decline aligns with the typical “citation lag” phenomenon in bibliometrics, as recently published articles require time to accumulate citations. Overall, these trends demonstrate that macrophage-mediated immune regulation has become a rapidly expanding frontier in IVDD research. We anticipate this field will maintain a robust growth trajectory in the coming years.

### 3.2 Distribution and collaboration networks of countries and institutions

**Figure 3:**
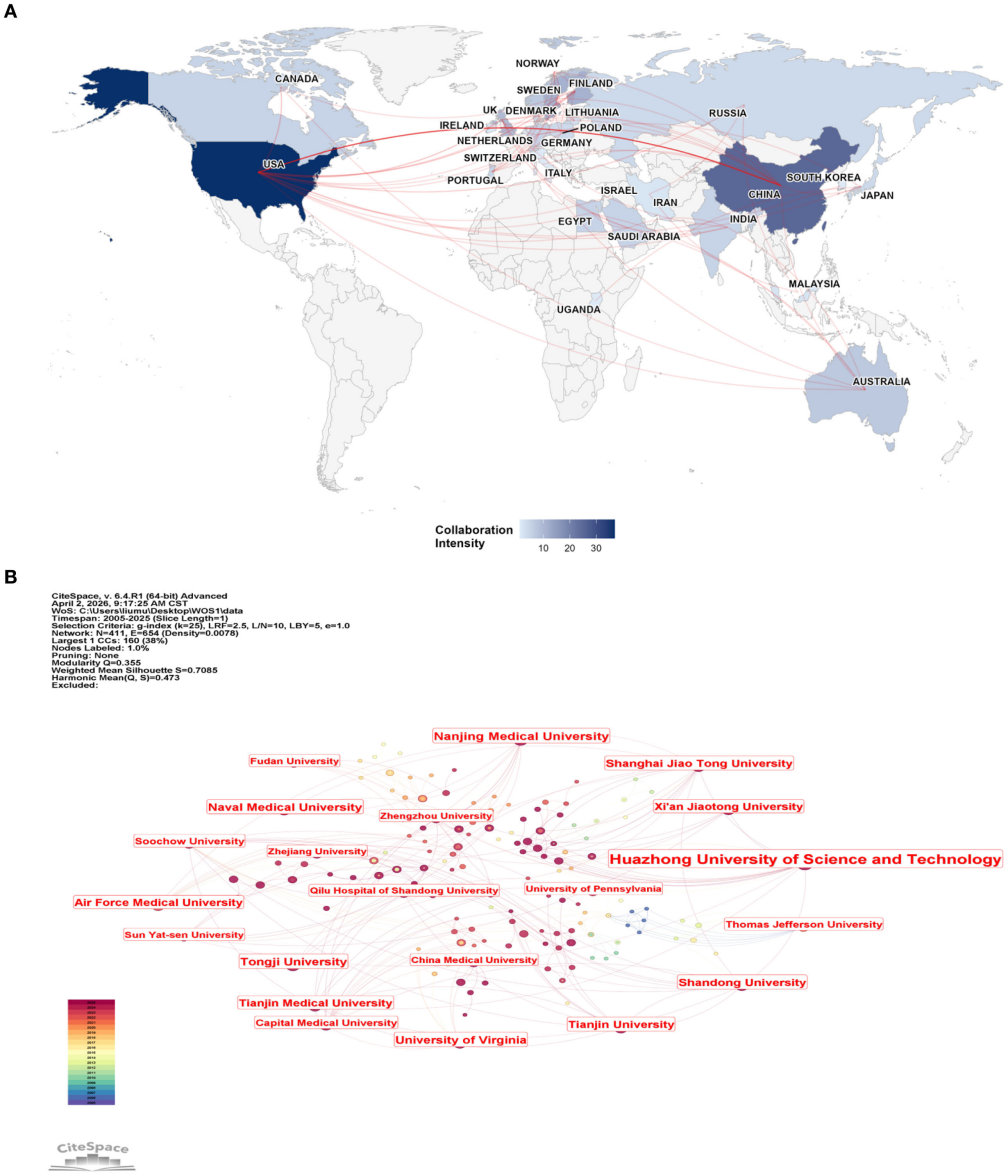
Collaboration networks of countries and institutions in macrophage-related research IVDD. **(A)** The geographical distribution and collaborative relationships of contributing countries. The depth of the blue color indicates the collaboration intensity, and the red connecting lines represent the collaborative links between different countries. **(B)** The institutional collaboration network map. Each node represents an institution, with larger nodes indicating a higher volume of publications. The lines connecting the nodes illustrate the collaborative relationships between these institutions.

A visual analysis of contributing countries and institutions reveals the global distribution of academic output and collaboration dynamics in this field. As shown in **Table 1** and **Figure 3A**, academic output is heavily concentrated geographically. China ranks first with 450 publications, followed by USA with 140 publications. Together, these two nations constitute the primary research forces in this domain. They are followed by Japan (28 publications), South Korea (28 publications), and the United Kingdom (17 publications). Furthermore, the country collaboration network (**Figure 3A**) indicates that high-frequency partnerships occur predominantly among major publishing nations. While the most robust academic collaboration exists between China and the US (indicated by thick connecting lines), transnational collaborations among other countries remain relatively limited.

**Table 1:** The top 10 most productive countries and institutions in macrophage-related research in IVDD.

Rank	Countries	Article counts	Rank	Institutions	Country	Article counts	Centrality
1	China	450	1	Huazhong University of Science and Technology	China	47	0.06
2	USA	140	2	Korea University	South Korea	34	0
3	Japan	28	3	Shanghai Jiao Tong University	China	16	0.07
4	South Korea	28	4	Soochow University	China	15	0.07
5	United Kingdom	17	5	Nanjing Medical University	China	13	0.06
6	Canada	17	6	Sun Yat-sen University	China	13	0.04
7	Germany	16	7	University of Virginia	USA	12	0.01
8	Switzerland	11	8	Naval Medical University	China	12	0
9	Netherlands	10	9	Tongji University	China	11	0.01
10	Portugal	9	10	Shandong University	China	10	0.06

Regarding institutional distribution, Huazhong University of Science and Technology ranks first with 47 publications. It is followed by Korea University (34 publications) and Shanghai Jiao Tong University (16 publications). Notably, eight of the top ten most productive institutions globally are located in China, highlighting the nation’s immense activity in this research niche. However, the institutional collaboration network (**Figure 3B**) and betweenness centrality metrics reveal structural limitations in the current research architecture. Data from **Table 1** show that the betweenness centrality for all top ten institutions is consistently below 0.10 (maximum value: 0.07). Additionally, network clustering indicates that inter-institutional connections are largely confined to domestic nodes. This points to the lack of an extensive, multicenter, multinational collaboration network among core global institutions. Because most teams currently operate within relatively independent domestic clusters, multilateral international academic exchanges will require significant strengthening in the future.

### 3.3 Core author distribution and academic collaboration networks

Regarding author productivity (**Table 2**), Joo Han Kim ranks first with 21 publications, followed by Zengwu Shao (20 publications) and Hyuk Choi (17 publications). In terms of citation impact, Zengwu Shao leads the top ten most productive authors with a total of 503 citations. This highlights that his team’s research findings command high academic attention and provide significant reference value within the field.

**Table 2:** The top 10 most productive and most frequently co-cited authors in macrophage-related research in IVDD.

Rank	Author	Article counts	Citations	Co-cited author	Co-citation
1	kim, joo han	21	475	risbud, mv	269
2	shao, zengwu	20	503	hoyland, ja	177
3	choi, hyuk	17	305	iatridis, jc	176
4	jin, li	13	261	shapiro, im	163
5	xiao, li	13	261	boos, n	162
6	li, bin	13	369	zhang, y	137
7	hwang, min ho	12	267	freemont, aj	126
8	shin, jae hee	12	267	le maitre, cl	123
9	lv, xiao	12	298	wang, y	121
10	wang, huan	12	214	zhang, s	114

**Figure 4:**
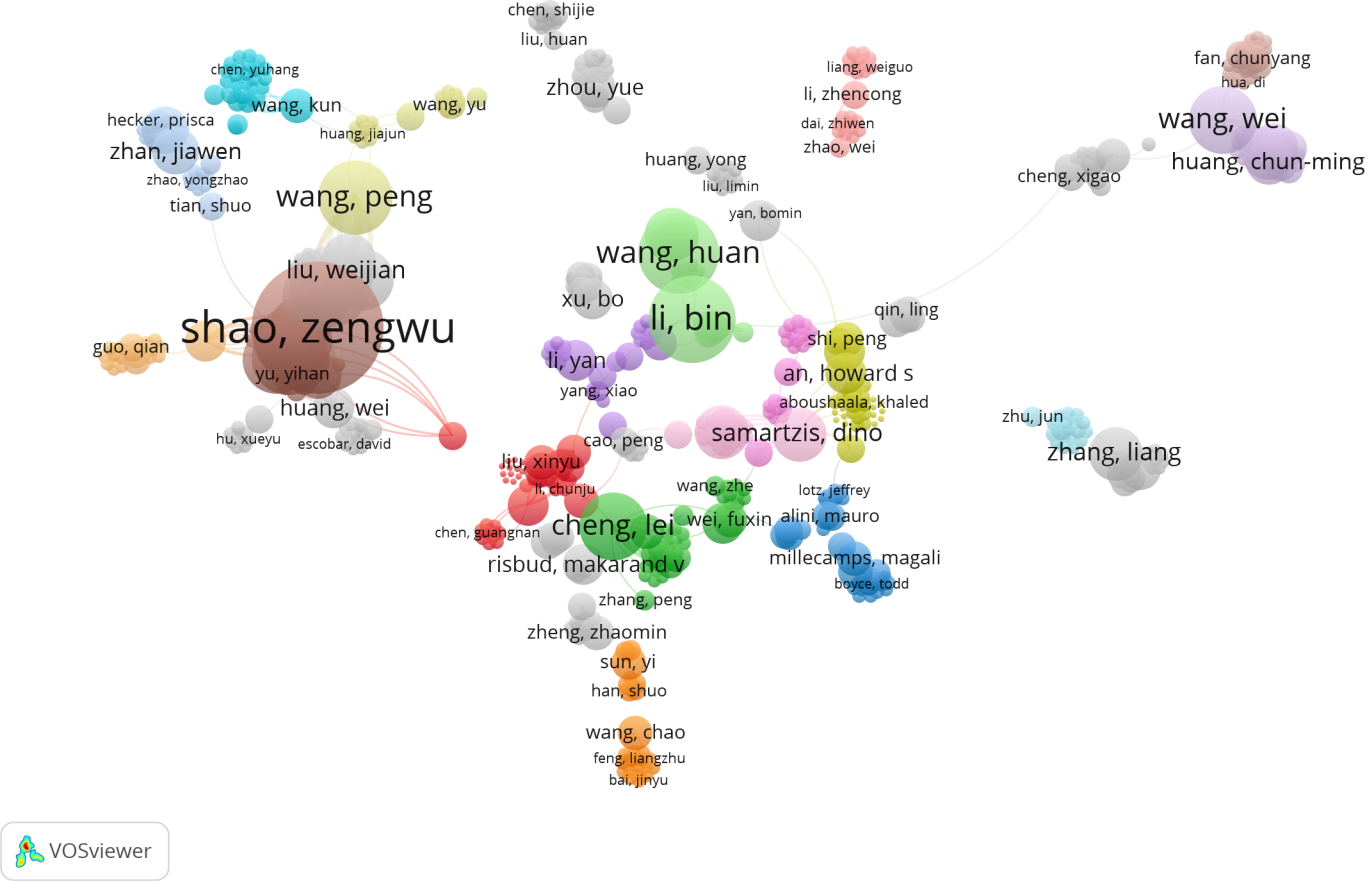
Co-authorship network of authors in macrophage-related research in IVDD. Each node represents an individual author, with the size of the node proportional to their total number of publications. The links between nodes indicate collaborative relationships, and the different colors signify distinct clusters of closely collaborating researchers.

The author co-occurrence network (**Figure 4**) illustrates the academic collaboration structure within this domain. The network features several relatively independent clusters, such as a prominent node group centered around Zengwu Shao and another central cluster including scholars like Huan Wang, Bin Li, and Lei Cheng. Topological features reveal dense connections among nodes within the same cluster, reflecting close collaboration within established research teams or affiliated institutions. However, connections between the core nodes of different clusters remain sparse. Consistent with our institutional collaboration analysis, these findings suggest that current research is predominantly led by isolated teams. A deep, cross-team scientific collaboration network has yet to be fully established.

A co-cited author analysis helps delineate the intellectual base of the field. As shown in **Table 2**, MV Risbud leads significantly with 269 co-citations, followed by JA Hoyland (177 citations) and JC Iatridis (176 citations). Notably, there is a distinct separation between highly productive authors and highly co-cited authors. The highly co-cited scholars predominantly focused on the classical pathology and mechanotransduction mechanisms of IVDD, forming the early theoretical foundation of the field. In contrast, the most productive current authors primarily concentrate on the emerging intersection of macrophage polarization and immune regulation. This distribution pattern reflects the intellectual evolution of IVDD research, which has transitioned from a traditional clinical orthopedic perspective to a modern, interdisciplinary focus on immunology and metabolism.

### 3.4 Core journals and interdisciplinary knowledge flow analysis

As shown in **Table 3**, *Frontiers in Immunology* (34 publications), *Spine* (22 publications), and the *International Journal of Molecular Sciences* (21 publications) are the top three most productive journals in this field. These leading journals predominantly belong to the JCR Q1 quartile and cover the disciplines of immunology, orthopedics, and cell biology. This distribution indicates that this interdisciplinary research has garnered broad recognition across multiple scientific domains. In the co-cited journal analysis, *Spine*—a traditional spinal surgery journal—ranks first with 1,189 co-citations, representing the core clinical and pathological foundation of the field. Concurrently, *Frontiers in Immunology* not only leads in publication volume but also ranks sixth in co-citations (276). This dual prominence demonstrates its central role in both disseminating cutting-edge literature and building the theoretical framework for immunological mechanisms in IVDD.

**Table 3:** The top 10 most productive and most frequently co-cited journals in macrophage-related research in IVDD.

Rank	Journal	Article counts	IF	JCR(2024)	Rank	Co-cited Journal	Citation	IF	JCR(2024)
1	Frontiers in Immunology	34	5.9	Q1	1	Spine	1189	3.5	Q1
2	Spine	22	3.5	Q1	2	Journal of Orthopedic Research	347	2.3	Q2
3	International Journal of Molecular Sciences	21	4.9	Q1	3	Spine Journal	310	4.7	Q1
4	Jor Spine	20	3.9	Q1	4	Osteoarthritis and Cartilage	286	9	Q1
5	Osteoarthritis and Cartilage	19	9	Q1	5	European Spine Journal	280	2.7	Q2
6	Scientific Reports	19	3.9	Q1	6	Frontiers in Immunology	276	5.9	Q1
7	Spine Journal	17	4.7	Q1	7	International Journal of Molecular Sciences	265	4.9	Q1
8	Advanced Science	17	14.1	Q1	8	Arthritis Research & Therapy	253	4.6	Q1
9	Journal of Orthopedic Research	16	2.3	Q2	9	Scientific Reports	219	3.9	Q1
10	Frontiers in Cell and Developmental Biology	15	4.3	Q1	10	Nature Reviews Rheumatology	196	32.7	Q1

**Figure 5:**
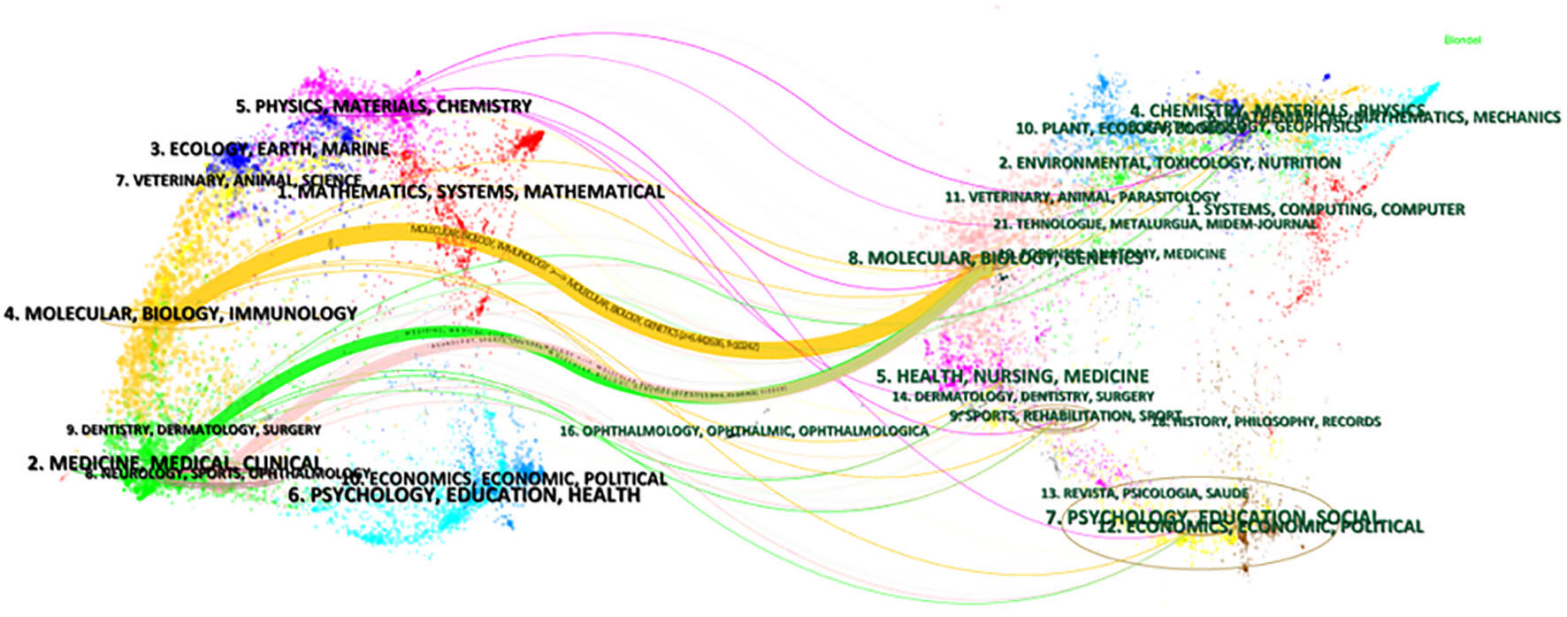
A dual-map overlay of journals in macrophage-related research in IVDD. The citing journals are located on the left, representing the research frontiers, while the cited journals are on the right, representing the intellectual knowledge base. The colored curves represent the citation pathways, illustrating the flow of knowledge.

The dual-map overlay of journals (**Figure 5**) illustrates the trajectory of interdisciplinary citations, displaying citing journals on the left and cited journals on the right. The map reveals two primary citation pathways. First, the yellow pathway indicates that research published in “molecular, biology, immunology” journals primarily cites foundational literature from “molecular, biology, genetics” journals. Second, the green pathway shows that research in “medicine, medical, clinical” journals relies heavily on basic theories from both the “molecular, biology, genetics” and “health, nursing, medicine” domains. This citation trajectory confirms that the research focus in this field has transcended the traditional scope of clinical orthopedics. Instead, it exhibits a deep, multidisciplinary integration of clinical medicine, molecular biology, and immunology.

### 3.5 Keyword co-occurrence and research hotspot evolution analysis

**Figure 6:**
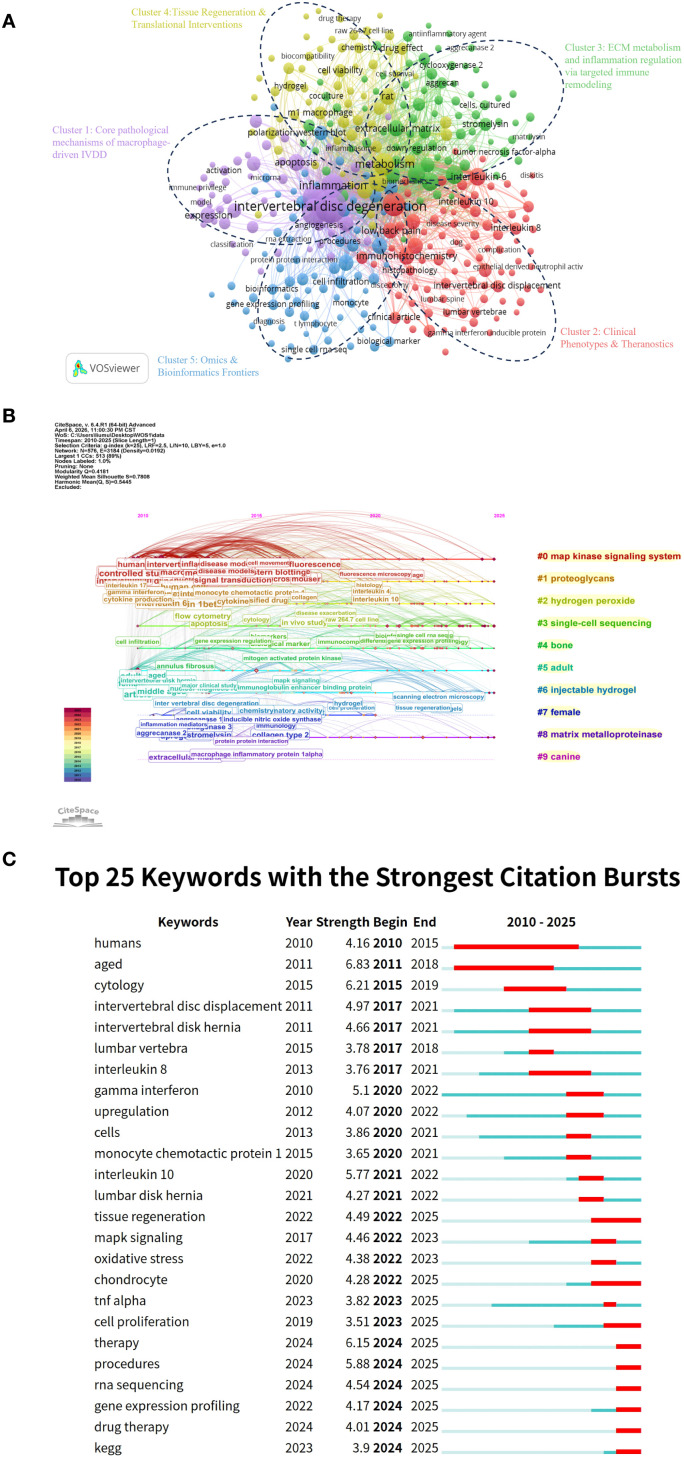
Keyword analysis of macrophage-related research in IVDD. **(A)** Co-occurrence network map of keywords. The nodes are grouped into five distinct clusters (indicated by different colors and dashed boundaries) representing the main research themes. **(B)** Timeline visualization of keyword clusters, illustrating the historical evolution and developmental span of different research topics. **(C)** The top 25 keywords with the strongest citation bursts. The red bars represent the active duration of the citation burst for each specific keyword.

Keyword co-occurrence and clustering analyses reveal the thematic architecture of the field. As shown in **Table 4**, high-frequency keywords are concentrated around “nucleus pulposus,” “inflammation,” “metabolism,” and related cytokines (e.g., interleukin-1 beta and tumor necrosis factor). This suggests that microenvironmental inflammatory cascades and metabolic imbalances are the core directions of investigation. Based on the keyword co-occurrence network (**Figure 6A**), research themes can be categorized into five core clusters: macrophage-driven IVDD pathological mechanisms (purple); extracellular matrix (ECM) metabolism and inflammatory regulation under targeted immune intervention (green); tissue regeneration and translational intervention (yellow); multi-omics and bioinformatics applications (blue); and clinical phenotypes and diagnostics (red). These clusters comprehensively cover the primary research framework, ranging from clinical pathology and foundational micro-mechanisms to biomaterial-based translational interventions.

**Table 4:** The top 10 most frequently occurring keywords in macrophage-related research in IVDD.

Rank	Keyword	Occurrences
1	intervertebral disc degeneration	548
2	macrophage	323
3	nucleus pulposus	247
4	inflammation	233
5	intervertebral disc	229
6	metabolism	205
7	pathology	149
8	protein expression	130
9	interleukin-1 beta	129
10	tumor necrosis factor	128

The keyword timeline (**Figure 6B**) and citation burst analysis (**Figure 6C**) demonstrate the temporal evolution of research hotspots. **Figure 6C** shows that the early stage of the field (2010–2020) primarily focused on basic phenomenological observations, highlighting terms such as “humans,” “cytology,” and chemokines (e.g., “monocyte chemotactic protein 1”). Since 2021, keywords including “oxidative stress,” “RNA sequencing,” and “gene expression profiling” have exhibited strong burst strengths. This indicates a shift in mechanistic research from single-factor exploration toward high-throughput, global molecular network analyses. Simultaneously, “drug therapy” and “tissue regeneration” have emerged as currently active burst terms. These evolutionary trends suggest a clear, two-pronged research frontier. First, researchers are increasingly utilizing multi-omics sequencing to elucidate complex disease mechanisms. Second, they are developing biomaterials to target macrophage polarization, aiming to restore microenvironmental homeostasis and promote tissue regeneration.

### 3.6 Core cited references and citation burst analysis

**Figure 7:**
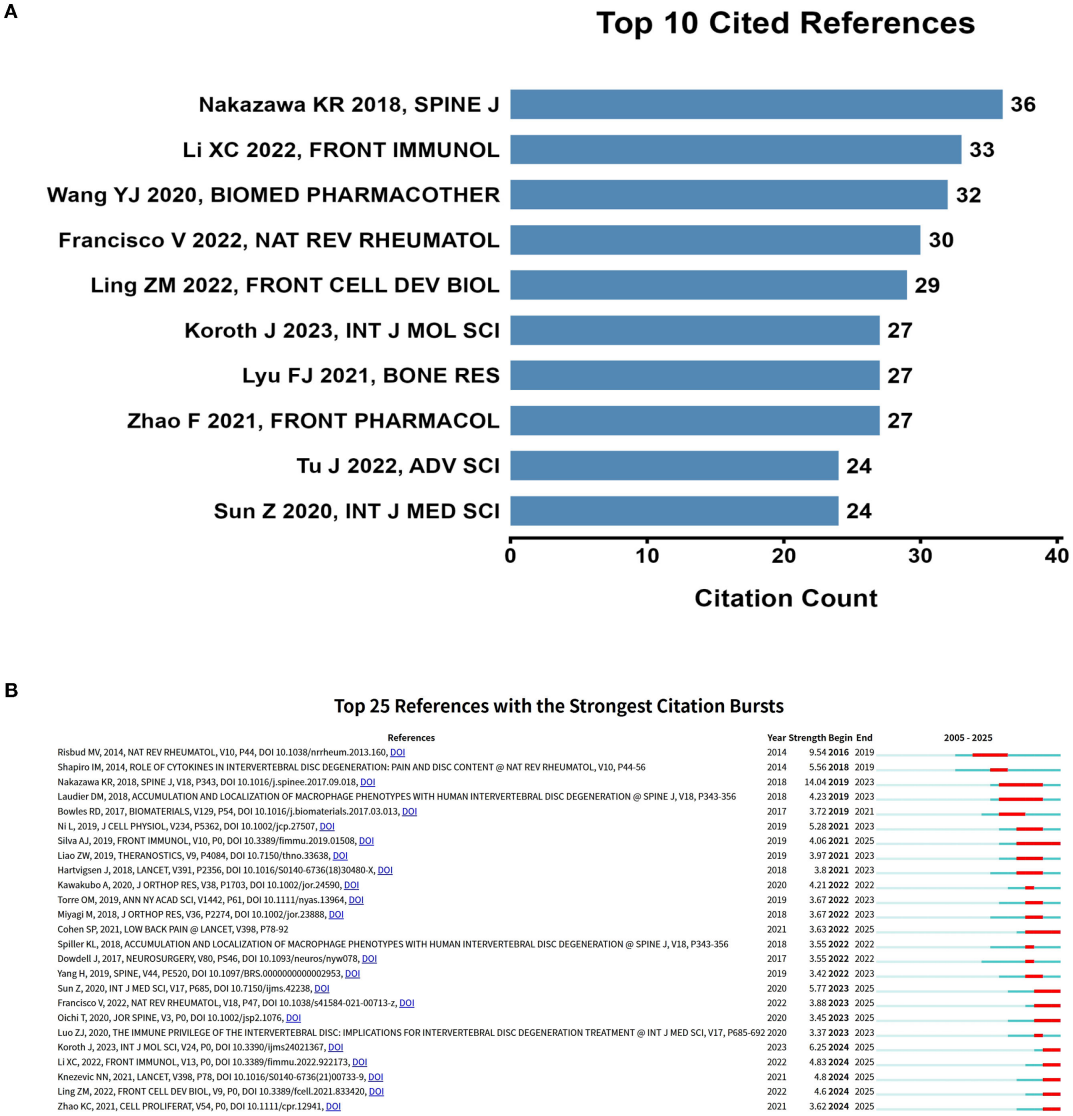
Citation analysis of references related to macrophages in IVDD. **(A)** The top 10 most cited references in the field. **(B)** The top 25 references with the strongest citation bursts. The red segments indicate the time interval during which a reference experienced a citation burst.

An analysis of highly cited references reveals the core literature foundation of the field (**Figure 7A**). Among the top ten co-cited references, a study by Nakazawa et al. (2018), published in *The Spine Journal*, ranks first with 36 citations (20). This study investigated the accumulation characteristics of different polarized macrophage phenotypes in degenerative intervertebral disc tissues, providing a crucial pathological basis for analyzing the immune microenvironment. Ranking second is an article by Li et al. (2022), published in *Frontiers in Immunology* (33 citations) (21). This work focuses on the regulatory mechanisms of immune homeostasis imbalance and macrophage polarization during disc degeneration. Additionally, a review by Francisco et al. (2022) in *Nature Reviews Rheumatology* ranks fourth, systematically introducing a novel “immunometabolic” perspective on IVDD (22). The shift in highly cited sources from traditional surgical journals to those focused on immunology and molecular biology is notable. It reflects the deepening of IVDD research, which has expanded beyond classical biomechanics into the complex intersection of microenvironmental metabolism and immune regulation.

A citation burst analysis (**Figure 7B**) highlights shifting academic focal points over time. Among the top 25 references with the strongest citation bursts, early research (bursts beginning in 2016–2019) primarily focused on the disruption of disc homeostasis by local cytokines and inflammatory responses. This era is well represented by Risbud et al.’s review on cytokine mechanisms (23). Since 2021, a new wave of references with high burst strengths has emerged. Studies by Francisco et al. (2022), Li et al. (2022), and Koroth et al. (2023) exemplify this recent surge, with active burst periods extending into 2025 (21, 22, 24). Current hotspots focus heavily on the regulatory networks of macrophage polarization, signal crosstalk between immune cells and nucleus pulposus cells, and targeted tissue engineering strategies for microenvironmental reconstruction. Ultimately, these citation trends confirm that the targeted regulation of macrophage phenotypes and immune microenvironment remodeling constitute the current research frontier for both foundational mechanistic exploration and translational medicine in IVDD.

## 4 Discussion

### 4.1 General overview

This study utilized bibliometrics to systematically review the landscape of macrophage-related research in IVDD from 2005 to 2025. Trend analysis indicates a significant surge in publications since 2021 (**Figure 2**), corroborating a substantial paradigm shift in the field’s research focus. As pathological explorations have deepened, the academic consensus regarding IVDD pathogenesis has gradually evolved from the traditional “mechanical wear” hypothesis to the “immune microenvironment imbalance” theory (25). Furthermore, recent Mendelian randomization analyses and transcriptomic evidence reveal that the local infiltration of peripheral immune cells and their interaction with the degenerative microenvironment causally drive IVDD progression (26). As pivotal regulatory cells of the innate immune system, macrophages play a central role in microenvironmental remodeling. This central role establishes a strong theoretical foundation for targeting macrophages in precision immunotherapy strategies (27).

Regarding scientific output and collaboration networks, China and USA represent the primary research forces in this field. As shown in **Table 1**, **Figure 3**, China dominates in both total publication volume and the number of highly productive institutions. This dominance is likely due to the country’s sustained research investment in biomedical materials, targeted delivery systems, and orthopedic immunology in recent years. However, institutional collaboration networks and betweenness centrality metrics reveal significant limitations within the current research ecology. Despite high overall publication volumes, the betweenness centrality of core, highly productive institutions remains consistently low (all < 0.10). Moreover, academic collaborations are predominantly confined to domestic networks. This regional clustering indicates that research teams largely operate through independent, internal partnerships. A primary barrier to expanding these networks internationally is the difficulty of aligning localized research methodologies to address the highly heterogeneous nature of IVDD pathology. Without unified experimental pipelines, the cross-validation of complex immune microenvironment data remains challenging.

Overcoming this structural fragmentation requires targeted, collaborative strategies. The field must prioritize the establishment of robust, multinational, and multicenter partnerships among top-tier institutions. By strengthening multilateral international academic exchanges, global researchers can consolidate fragmented data, ultimately accelerating the development and clinical translation of standardized immune intervention protocols.

### 4.2 Intellectual base of macrophage-related research in IVDD

The intellectual base of any scientific discipline is primarily constructed by its core co-cited journal clusters and high-impact co-cited literature. As demonstrated by our dual-map overlay of journals and co-citation network analysis (**Figure 5**, **Table 3**), the intellectual base of macrophage research in IVDD is undergoing a distinct interdisciplinary evolution. The early theoretical foundation relied heavily on traditional orthopedic journals, particularly *Spine*. Through classical histopathological observations, these early studies confirmed the infiltration of macrophages and the enrichment of pro-inflammatory cytokines within degenerative disc tissues. Ultimately, these findings established the initial clinical and pathological framework for the field.

Driven by the advancement of single-cell omics technologies and molecular immunology, this knowledge framework has expanded beyond a traditional anatomical focus. It now deeply encompasses molecular biology, epigenetics, and the intricate network regulation of the immune microenvironment. Furthermore, our citation burst analysis (**Figure 7B**) reveals that recent research has shifted from merely observing inflammatory phenomena to investigating the mechanistic regulation of complex cellular and molecular networks.

Currently, the intellectual base of this field focuses on two primary dimensions. First, researchers are exploring the molecular driving mechanisms of macrophage polarization and the resulting M1/M2 phenotype imbalance. This includes investigating the altered expression of specific transcription factors and the activation of immune receptor pathways (10). Second, there is a strong emphasis on the intercellular communication between infiltrating macrophages, intrinsic nucleus pulposus cells (NPCs), and the extracellular matrix (ECM) (28). This aberrant paracrine immune crosstalk has been shown to directly induce ECM degradation and cell apoptosis, creating a pathogenic positive feedback loop that accelerates disc degeneration. Ultimately, novel mechanistic research into immunometabolism and cellular crosstalk has provided a robust theoretical basis for emerging tissue engineering strategies centered on targeted macrophage reprogramming.

### 4.3 Research hotspots and frontiers

Keyword co-occurrence and burst analyses objectively reflect the research trajectories and evolutionary hotspots within this field. The keyword burst map (**Figure 6C**) identifies “gamma interferon” and “monocyte chemotactic protein 1” as early mechanistic keywords with high burst strengths. These terms highlight the critical role of local chemokine networks in early pathological research, where studies focused on how pro-inflammatory factors drive peripheral macrophage infiltration and disrupt microenvironmental homeostasis. Furthermore, as shown in **Figures 6B**, **C**, technological iterations over the past three years have shifted core research clusters and high-strength burst keywords toward “single-cell sequencing,” “oxidative stress,” “injectable hydrogels,” and “tissue regeneration.” This suggests that mechanistic analyses relying on multi-omics technologies and targeted interventions using functional biomaterials constitute the current core frontiers of this field.

**Figure 6A** visually displays five core research clusters derived from the keyword co-occurrence network analysis. These clusters comprehensively reflect the primary academic framework for studying macrophages within the intervertebral disc immune microenvironment, encompassing everything from clinical pathological phenomena to microscopic molecular mechanisms. The five clusters are: Purple Cluster: Macrophage-driven pathological mechanisms of IVDD,Green Cluster: Targeted immune regulation and extracellular matrix (ECM) metabolic remodeling, Yellow Cluster: Tissue regeneration and translational interventions, Blue Cluster: Multi-omics and bioinformatics analyses, Red Cluster: Clinical phenotypes and diagnostic associations.

(I) Macrophage-Driven IVDD Pathological Mechanisms: In recent years, research on the macrophage-mediated degenerative microenvironment has deepened. The focus has shifted from macroscopic observations of inflammatory phenotypes to the molecular mechanisms of intercellular communication and programmed cell death. Chemokine networks and oxidative stress constitute critical nodes driving this pathological evolution. Local hypoxia, hyperosmolarity, and the accumulation of endogenous ROS in the degenerative NP microenvironment can induce the chemotactic infiltration of peripheral monocytes/macrophages, promoting their polarization toward the M1 phenotype.

A bidirectional pathological crosstalk exists between infiltrating macrophages and intrinsic NPCs. Damaged NPCs secrete microRNA-enriched exosomes (e.g., miR-27a-3p) that induce M1 polarization in peripheral macrophages, thereby amplifying the inflammatory cascade (29). Conversely, ROS stimulation hyperactivates NLRP3 inflammasomes within these M1 macrophages. This activation releases IL-1β and IL-18, which further accelerate NPC pyroptosis (30). At the epigenetic level, omics analyses indicate that the aberrant expression of DNMT1 can directly drive M1 polarization by suppressing SIRT6. This highlights the regulatory role of epigenetic modifications in microenvironmental imbalances (31). Targeting specific signal receptor axes provides a viable intervention strategy against this pathogenic network. For instance, activating the CX3CL1/CX3CR1 axis can induce macrophage reprogramming toward the M2 phenotype, antagonizing NPC apoptosis via paracrine effects (32). Meanwhile, prosaposin (PSAP) can regulate intercellular metabolic homeostasis through the GPR37 receptor system (33).

(II) ECM Metabolism and Inflammatory Regulation via Targeted Immune Remodeling: The loss of ECM homeostasis is the primary pathological driver of biomechanical failure in the intervertebral disc. The high co-occurrence of the nodes “metabolism” and “inflammation” in the keyword map indicates that specific macrophage phenotypes play a key role in matrix degradation. Recent mechanistic studies highlight the molecular regulatory roles of local iron dyshomeostasis and ferroptosis in macrophage-mediated ECM remodeling. The accumulation of lipid peroxides and free iron overload in the degenerative microenvironment not only induces lipotoxicity in NPCs but also reprograms macrophage metabolic pathways. This accelerates collagen scaffold degradation by upregulating MMPs (34).

The M1-dominant, pro-inflammatory microenvironment can disrupt the iron homeostasis of intrinsic NPCs. By depleting GSH to induce massive ROS generation, this disruption produces a synergistic damaging effect alongside the ferroptosis pathway, thereby exacerbating tissue destruction (35). Furthermore, classical inflammatory pathways serve as molecular hubs connecting macrophage polarization and ECM degradation. For example, high expression of the zinc transporter ZIP8 can activate the Wnt/β-catenin signaling pathway. This amplifies the secretion of inflammatory mediators while promoting M1 polarization, ultimately leading to the disintegration of the ECM structure (36). Pharmacological interventions targeting relevant metabolic markers demonstrate substantial matrix-protective potential. Quercetin, for instance, can competitively disrupt the assembly of the CEBPB-HMGA1 transcriptional complex, blocking the release of macrophage-derived TNF-α and decelerating enzymatic ECM degradation (37). Concurrently, the introduction of high-throughput single-cell impedance flow cytometry has enabled the single-cell-level monitoring of macrophage phenotypes and metabolic fluxes in *ex vivo* microenvironments, providing vital technical support for the high-throughput screening of targeted drugs (38).

(III) Regeneration Repair and Translational Medicine Intervention: The significant citation bursts of “injectable hydrogels” and “tissue regeneration” indicate that immunomodulatory strategies utilizing biomedical materials have become a crucial research direction for reversing the IVDD microenvironment. Traditional local, passive anti-inflammatory therapies are evolving into intelligent delivery systems endowed with microenvironmental responsiveness and biological activity. Consequently, the design philosophy has shifted from providing purely physical mechanical support to actively reconstructing immune homeostasis.

For example, a composite hydrogel system loaded with EGCG can provide mechanical support while simultaneously inducing local M2 macrophage polarization, inhibiting pro-inflammatory pathways, and promoting matrix metabolic balance (39). To address persistent oxidative stress, self-assembling biomimetic hydrogels with antioxidant enzyme (e.g., superoxide dismutase) activity have been proven to scavenge local ROS, alleviate NPC senescence, and induce an anti-inflammatory macrophage phenotype conversion (40). Additionally, ROS/pH dual-responsive hydrogels have achieved the spatially controlled release of drugs through a “sense-and-release” mechanism. Their active ingredients can target and inhibit the PI3K/AKT/NF-κB signaling axis, suppressing cell apoptosis and ECM degradation (41). Regarding long-term immunomodulation, PLGA sustained-release microsphere composite scaffolds loaded with interleukin-4 (IL-4) can maintain a prolonged anti-inflammatory microenvironment while inducing M2 polarization. This promotes the repair of the type II collagen network *in vivo* (42). Targeted hydrogel platforms that integrate the dual functions of inhibiting M1 activation and promoting M2 conversion provide a material optimization pathway to resolve the diminishing efficacy often observed with single-cytokine therapies (43).

Therapeutically, these hotspots translate into precision immunomodulation and advanced delivery systems. Modulating macrophage-NPC crosstalk provides a direct intervention target. For example, macrophage-derived PDGF-BB maintains NPC glycolysis to inhibit TXNIP-NLRP3 inflammasome assembly and prevent pyroptosis (44). Sustaining such interventions within the avascular disc requires highly functionalized biomaterials. Decellularized ECM hydrogels enable the continuous release of M2 macrophage-derived small extracellular vesicles (M2-sEVs), utilizing exosomal miR-221-3p to suppress the PTEN/NLRP3 pyroptosis pathway *in vivo* (45). Furthermore, integrating lipid nanoparticles (LNPs) into composite scaffolds advances targeted gene therapy. Biomimetic GelMA/fucoidan hydrogel microspheres grafted with LNPs can efficiently deliver Bry mRNA to NPCs. The sustained release of fucoidan dampens local inflammation by downregulating COX2 and iNOS, while Bry translation directly restores ECM synthesis (46). Ultimately, these integrated strategies shift the clinical focus from passive symptom management to active microenvironmental remodeling.

(IV) Multi-omics and Bioinformatics Exploration: With the advancement of high-throughput sequencing technologies, the functional profiling of macrophages in IVDD has progressed to the single-cell and spatial multi-omics levels. Traditional bulk RNA sequencing struggles to capture the heterogeneity of immune cells; however, scRNA-seq has revolutionized the academic understanding of dynamic microenvironmental evolution. Using scRNA-seq, researchers constructed a single-cell atlas of the human degenerative NP, identifying transitional macrophage subpopulations that exert specific functions at different stages of degeneration (47). By comparing the transcriptomes of normal and degenerative tissues, studies have revealed how macrophage subpopulations mediate NPC apoptosis or calcification processes through specific ligand-receptor interaction networks (48). In exploring the therapeutic mechanisms of MSCs, scRNA-seq identified a novel macrophage reparative subpopulation induced by MSC paracrine factors, elucidating its immunomodulatory mechanisms at a single-cell resolution (49).

Furthermore, spatial transcriptomics has effectively compensated for the lack of 3D anatomical spatial localization inherent to scRNA-seq. Studies have mapped the spatial transcriptome of the intervertebral disc, successfully localizing NP progenitor cells and their surrounding immune microenvironment (50). When combined with multi-omics analyses, this research confirms the vital spatial role of signaling axes, such as SPP1-CD44, in regulating local immune polarization and matrix remodeling (51).

(V) Clinical Phenotypes and Diagnostic Translation: Translating macrophage polarization theory into clinical interventions remains the ultimate objective of this field. Large clinical sample and histological studies suggest that immune microenvironment imbalances exist not only in the intervertebral disc proper but also extend to the cartilage endplate and paraspinal muscle tissues. This extensive involvement constitutes the pathological basis of discogenic pain.

As a typical imaging feature of IVDD, MCs highly correlate with the degree of local immune inflammation. Analyses of human surgical tissues show that the expression levels of GM-CSF and IL-1β are significantly elevated in tissues with Type II MCs, confirming the correlation between macrophage activity and the severe pain phenotype (52). In the endplate microenvironment, macrophage MIF and its receptor CD74 are abnormally highly expressed in tissues with Type I MCs. The activation of this pathway further amplifies osteoclastogenesis and inflammatory cascade effects (53). Furthermore, studies on lumbar disc herniation complicated by endplate avulsion reveal that extruded cartilage components mechanically restrict the chemotactic infiltration of macrophages and impede neovascularization. This lowers the spontaneous resorption rate of herniated tissues, thereby prolonging radicular pain (54).

Degeneration-induced immune cascades also exhibit anatomical diffusion. Evidence confirms that local pro-inflammatory signals can spread to the adjacent multifidus muscles, upregulating the proportion of M1 macrophages. This induces fat infiltration and muscle fiber atrophy, providing an immunological explanation for the clinical back muscle weakness often accompanying degeneration (55). Finally, the integration of single-cell and multi-omics sequencing provides a critical foundation for non-invasive IVDD diagnostics. At the tissue level, high-resolution transcriptomics identifies fibrotic late-stage NPCs as a specific pathogenic subset. These cells overexpress SRGN to drive macrophage infiltration and M1 polarization, establishing SRGN as a viable biomarker for early structural failure (56). At the systemic level, combining scRNA-seq with bulk RNA data reveals TNFAIP6 and COL6A2 as robust blood-based signatures capable of distinguishing severe from minor IVDD (57). Together, these distinct marker panels facilitate the development of clinical liquid biopsies, allowing clinicians to detect local inflammatory activity well before irreversible morphological changes become visible on MRI.

## 5 Future research trends

Bibliometric forecasting indicates a definitive shift in the research landscape. Early studies primarily focused on macroscopic cytokine observations. The current epicenter prioritizes precise clinical phenotyping, intelligent regenerative materials, and single cell regulatory networks. Clinically, elucidating the pathological links among aberrant macrophage polarization, Modic changes, and adjacent tissue inflammation will enable the precise subtyping of discogenic pain. Therapeutically, the field is transitioning toward active microenvironmental remodeling. Future interventions will increasingly rely on advanced nanoscale delivery vectors. Integrating exosomes or synthetic LNPs into responsive composite hydrogels represents a frontier strategy. These smart platforms enable the sustained and localized delivery of therapeutic payloads, such as specific microRNAs or transcription factor mRNAs, to achieve targeted macrophage reprogramming. Mechanistically, future studies must further deconstruct the complex degenerative cascade. This effort includes exploring specific intercellular communication axes, mitochondrial metabolic disorders, and ferroptosis pathway activation. Integrating single cell sequencing with spatial transcriptomics will be crucial. This combined approach will accurately map the spatial heterogeneity and dynamic evolution of immune cells within the degenerative niche, ultimately guiding next generation precision immunotherapies.

## 6 Limitations

This bibliometric analysis presents certain objective limitations. First, literature data were retrieved exclusively from the WoSCC and Scopus databases. We excluded PubMed because its standard export records lack the comprehensive citation linkage matrices required by algorithms in VOSviewer and CiteSpace. However, we acknowledge that this exclusion introduces an inherent selection bias. PubMed and EMBASE provide exceptionally broad coverage of purely clinical and pharmacological literature. Omitting these databases may result in the underrepresentation of early-stage clinical trials, localized medical case reports, and specific translational studies. Furthermore, the exclusion of non-English databases (such as CNKI) restricts our findings to English-language publications, potentially omitting relevant domestic research. Future bibliometric analyses should utilize advanced APIs to merge disparate database formats, thereby mitigating these biases and capturing a more holistic clinical landscape.

## 7 Conclusion

This study utilized systematic bibliometrics and network visualization to quantitatively analyze the global landscape of macrophage research in IVDD over the past two decades. The results reveal a significant surge in publication volume. While core author networks and dominant research institutions have been established, multinational collaborative networks remain critically underdeveloped. Keyword clustering and citation burst analyses delineated five primary evolutionary frameworks: macrophage-driven pathological mechanisms, ECM metabolism and inflammatory regulation, tissue regeneration, multi-omics frontiers, and clinical phenotype diagnostics. These clusters highlight a definitive paradigm shift. Research has moved from early macroscopic cytokine observations to precise immunometabolic interventions. Currently, the integration of high resolution single cell transcriptomics and advanced drug delivery systems, such as lipid nanoparticles and functionalized composite hydrogels, represents the absolute research frontier. These technologies enable the targeted reprogramming of macrophage phenotypes to actively reverse the degenerative microenvironment. Moving forward, deepening interdisciplinary integration and establishing robust multicenter international collaborations will be essential to translate these mechanistic innovations into effective nonsurgical clinical therapies for discogenic pain.

## Data Availability

The original contributions presented in the study are included in the article/supplementary material. Further inquiries can be directed to the corresponding authors.

## Glossary

AKT: Protein kinase B
APIs: Application Programming Interfaces
Bry: Brachyury
CD44: Cluster of differentiation 44
CD74: Cluster of differentiation 74
CEBPB: CCAAT/enhancer-binding protein beta
CNKI: China National Knowledge Infrastructure
COL6A2: Collagen type VI alpha 2 chain
COX2: Cyclooxygenase-2
CX3CL1: C-X3-C motif chemokine ligand 1
CX3CR1: C-X3-C motif chemokine receptor 1
DAMPs: Damage-associated molecular patterns
dECM: Decellularized extracellular matrix
DNMT1: DNA methyltransferase 1
ECM: Extracellular matrix
EGCG: Epigallocatechin gallate
EMBASE: Excerpta Medica Database
EMEs: Exercise-mimetic exosomes
GBD: Global Burden of Disease
GelMA: Gelatin methacryloyl
GM-CSF: Granulocyte-macrophage colony-stimulating factor
GPR37: G protein-coupled receptor 37
GSH: Glutathione
HMGA1: High mobility group AT-hook 1
HMGB1: High mobility group box 1
IL-1β: Interleukin-1 beta
IL-4: Interleukin-4
IL-18: Interleukin-18
iNOS: Inducible nitric oxide synthase
IVDD: Intervertebral disc degeneration
JCR: Journal Citation Reports
LNPs: Lipid nanoparticles
M2-sEVs: M2 macrophage-derived small extracellular vesicles
MCs: Modic changes
MIF: Macrophage migration inhibitory factor
miR: microRNA
MMPs: Matrix metalloproteinases
MRI: Magnetic resonance imaging
MSCs: Mesenchymal stem cells
NF-κB: Nuclear factor kappa B
NLRP3: NOD-LRR and pyrin domain-containing protein 3
NP: Nucleus pulposus
NPCs: Nucleus pulposus cells
PDGF-BB: Platelet-derived growth factor-BB
PI3K: Phosphoinositide 3-kinase
PLGA: Poly(lactic-co-glycolic acid)
PSAP: Prosaposin
ROS: Reactive oxygen species
scRNA-seq: Single-cell RNA sequencing
SIRT6: Sirtuin 6
SPP1: Secreted phosphoprotein 1
SRGN: Serglycin
TNFAIP6: Tumor necrosis factor-inducible gene 6 protein
TNF-α: Tumor necrosis factor-alpha
TXNIP: Thioredoxin-interacting protein
USA: United States of America
WoSCC: Web of Science Core Collection
YLDs: Years lived with disability
ZIP8: ZRT/IRT-like protein 8.
